# Efficacy and safety of acupuncture as a complementary therapy for sepsis: A protocol of systematic review and meta-analysis

**DOI:** 10.1097/MD.0000000000018025

**Published:** 2019-11-27

**Authors:** Jin Xian, Ling Wang, Changyun Zhang, Jian Wang, Yushuo Zhu, Huijuan Yu, Xin Zhang, Qiwen Tan

**Affiliations:** a Affiliated Hospital of Shandong University of Traditional Chinese Medicine; b Shandong University of Traditional Chinese Medicine, Jinan, Shandong, China.

**Keywords:** acupuncture, sepsis, systematic review

## Abstract

**Introduction:**

Sepsis is a physiological, pathological, and biochemical syndrome caused by infection. Acupuncture may be useful for sepsis. This systematic review aims to assess the efficacy and safety of acupuncture as a complementary therapy for sepsis.

**Methods and analysis:**

We will search PubMed, EMBASE, the Cochrane Central Register of Controlled Trials, China National Knowledge Infrastructure (CNKI), Wan Fang Database, Chinese Biomedicine (CBM) database, VIP database, and TCM Literature Analysis and Retrieval Database from inception to October 31, 2019 to identify any eligible study. We include all randomized controlled trials (RCTs) without any limitation of blinding or publication language, exclude cohort studies and case reports. Two reviewers will independently select studies, extract and manage data. The primary outcomes include the mortality at 28 days, acute physiology, and chronic health evaluation II scores. The secondary outcomes include the tumor necrosis factor α (TNF-α) counts, interleukin 6 (IL-6) counts, interleukin 10 (IL-10) counts, procalcitonin (PCT), lactic acid, the level of T cell subsets (CD3+, CD4+, CD8+, CD4+/CD8+), monocytes of human leukocyte antigen DR (HLA-DR), C-reactive protein (CRP), the numeration of leukocyte, intra-abdominal pressure, and adverse events or reactions. Statistical analyses will be performed using the Review Manager V.5.3 and R packages Metafor. We will use the Cochrane risk of bias tool for randomized trials to assess the risk of bias of included studies.

**Ethics and dissemination:**

This study will not involve personal information. Ethical approval will not be required. We will publish the results in a peer-reviewed journal.

**PROSPERO Trial registration number:**

CRD42019141491

## Abstract 2

Supplemental Digital Content is available in the text

## 1 Introduction

Sepsis is a physiological, pathological, and biochemical syndrome caused by infection. The incidence of sepsis is increasing,^[1,2]^ which causes or contributes to between one-third and one-half of all deaths in hospitals in the United States,^[3]^ may be related to the ageing of the population. This problem is equally serious in China and varied according to socioeconomic indicators.^[4]^ Although the mortality rate of in-hospital sepsis has decreased in recent years,^[5]^ but it is based on spending a lot of money on health care.^[2,6]^ Sepsis remains the most common cause of death in critically ill patients.

A large number of secretions of inflammatory mediators such as cell injury, multiple organ dysfunction syndrome, and death, is the characterized in the initial stage of sepsis. With the progress of sepsis, the body may have experienced an immunosuppressive stage leading to an aggravation of inflammation.^[7]^ Therefore, the treatment of sepsis can not only anti-inflammatory and ignore the regulation of the immune system. Newer therapeutic interventions are aimed at inflammation, immunosuppression, and protein catabolism alone. However, successful treatment of sepsis patients with chronic critical illness and persistent inflammation-immunosuppression and catabolism syndrome may require more complementary approaches.^[8]^

Acupuncture is an important part of complementary therapy guide by the theory of traditional Chinese medicine. Several studies showed that acupuncture maybe benefit for sepsis.^[9,10]^ The benefit may be related to the fact that acupuncture can reduce inflammation and regulate immunity. Animal experiments have shown that acupuncture can regulate both non-specific immune function and specific immune function. The influence of acupuncture depends on controlling the production of immune cytokines (interleukin-1b [IL-1b], interleukin-2 [IL-2], interleukin-4 [IL-4], interleukin-6 [IL-6], interleukin-8 [IL-8], tumor necrosis factor α [TNF-α], interferon-γ (IFN-γ), and interleukin-17 [IL-17])^[11,12]^ and improve the ratio of CD4+T cells/CD8+T cells and modulate the synthesis.^[13]^ This immunomodulatory effect has also been demonstrated in clinical trials.^[14]^

According to the published research, there is a lack of high-quality evidence on acupuncture in the treatment of sepsis. Therefore, this systematic review aims to assess the effectiveness and safety of acupuncture as a complementary therapy for sepsis.

## 2 Methods

### 2.1 Study registration

This systematic review protocol has been registered in PROSPERO (registration number: CRD42019141491). This protocol has been checked with Preferred Reporting Items for Systematic review and Meta-Analysis Protocols (PRISMA-P) checklist.^[15]^ If the protocol is modified, we will describe the information in the final report.

### 2.2 Criteria for including studies in the review

#### 2.2.1 Types of studies

We will include all randomized controlled trials (RCTs) of acupuncture therapy for sepsis without any limitation of blinding or publication language, and we will also exclude cohort studies, case reports, and duplicate publications.

#### 2.2.2 Types of participants

Participants will be include who have been diagnosed with sepsis by these criteria: 2001 SCCM/ESICM/ACCP/ATS/SIS International Sepsis Definitions Conference,^[16]^ Surviving Sepsis Campaign: International guidelines for management of severe sepsis and septic shock: 2008/2012/2016/2018.^[17–20]^ The Third International Consensus Definitions for Sepsis and Septic Shock,^[21]^ Expert consensus on diagnosis and treatment of sepsis formulated by Emergency Medicine Committee of Chinese Society of Integrated Traditional Chinese and Western Medicine in 2013,^[22]^ Definition, diagnostic criteria, main points, and description of TCM syndrome diagnosis of sepsis (draft),^[23]^ guidelines for the treatment of severe sepsis / septic shock in China (2014).^[24]^ There is no restriction on age, sex, or ethnicity of the enrolled subjects.

#### 2.2.3 Types of interventions

We will include all RCTs which treatment group using acupuncture plus routine therapies and control group using routine therapies alone. Routine therapies include anti-infection, nutritional support, fluid management, mechanical ventilation, and other necessary therapies. The acupuncture treatment defined as needle at acupoints on the meridians, including manual acupuncture or electroacupuncture, excluding other acupuncture treatments such as ear acupuncture, scalp acupuncture, dry needling, acupressure.

#### 2.2.4 Types of outcomes

##### 2.2.4.1 The primary outcomes

The primary outcomes include the mortality at 28 days and acute physiology and chronic health evaluation II (APACHE II) scores.

##### 2.2.4.2 The secondary outcomes

The secondary outcomes include inflammatory index, immune index, and gastrointestinal function, such as the TNF-α counts, IL-6 counts, IL-10 counts, procalcitonin (PCT), lactic acid, the level of T cell subsets (CD3+, CD4+, CD8+, CD4+/CD8+), monocytes of human leukocyte antigen DR (HLA-DR), C-reactive protein (CRP), the numeration of leukocyte, intra-abdominal pressure, and adverse events or reactions.

### 2.3 Search methods

#### 2.3.1 Electronic searches

We will search PubMed (1966 to October 2019), the Cochrane Central Register of Controlled Trials (update to October 2019), EMBASE (1980 to October 2019), China National Knowledge Infrastructure (CNKI) (1979 to October 2019), Wan Fang Data (1980 to October 2019), VIP database (1989 to October 2019), Chinese Biomedical Database (1978 to October 2019) and TCM Literature Analysis and Retrieval Database (1949 to October 2019). The search strategy is available in appendix 1.

#### 2.3.2 Searching other resources

We will search the US National Institutes of Health Ongoing Trials Register, the WHO International Clinical Trials Registry Platform, Chinese Clinical Trial Registry and Google Scholar for any relevant ongoing or unpublished trials.

### 2.4 Data collection and analysis

#### 2.4.1 Selection of studies

**Figure 1:**
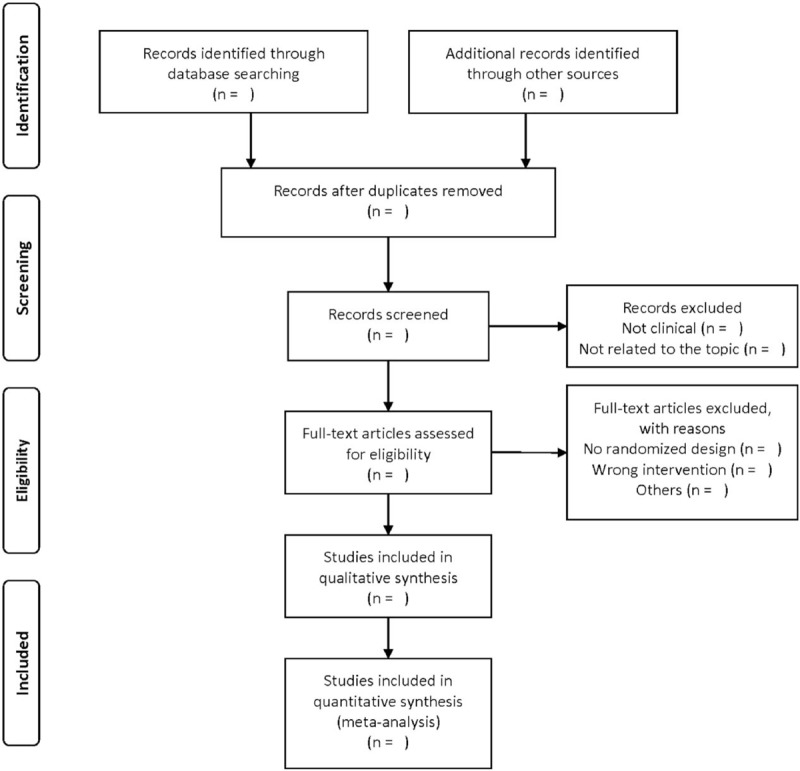
PRISMA flow diagram. PRISMA = Preferred Reporting Item for Systematic Review and Meta-analysis.

Two reviewers (CZ and YZ) will independently select the studies. They will check the results with each other. When disagreements occur, a third reviewer (XZ) will make the final decision. They will read the full texts of all included studies if necessary. Details of the selection process will be shown in the Preferred Reporting Items for Systematic Reviews and Meta-Analyses flow diagram (Fig. 1).^[25]^

#### 2.4.2 Data extraction and management

The citations will be screened independently by the 2 authors (LW and JX), they will extract the data using a standardized data extraction form and any differences of opinion between them will be resolved through discussion, if failed, by arbitration by the third reviewer (HY). We identified the following information for each trial: author's name, year of publication, inclusion and exclusion criteria, number of patients and reviews, type of acupuncture, and outcome measures.

#### 2.4.3 Risk of bias assessment

Two reviewers (JX and LW) will independently assess the risk of bias using the Cochrane risk of bias tool for randomized trials.^[26]^ They will compare their assessments, and discuss any differences in opinion between them, if this fails, through arbitration by a third reviewer (JW). Domains to be assessed: was there adequate sequence generation (selection bias)? Was allocation adequately concealed (selection bias)? Was knowledge of the allocated interventions adequately prevented during the study? Participants and personnel (performance bias) outcome assessors (detection bias); Were incomplete outcome data adequately addressed (attrition bias)? Are reports of the study free of suggestion of selective outcome reporting (reporting bias)? Was the study apparently free of other problems that could put it at risk of bias? The risk of bias for each domain will be graded high risk, low risk, and unclear risk of bias based on the relevant information extracted from each eligible study.

#### 2.4.4 Measures of treatment effect

We will conduct a meta-analysis if the studies can be combined. The risk ratio (RR) with 95% confidence intervals (CI) will be calculated for dichotomous data. Standardized mean difference (SMD) with 95% CI will be calculated for continuous data. We will provide a narrative synthesis of the outcomes and results of the studies if a meta-analysis is not possible.

#### 2.4.5 Dealing with missing data

We will try to contact the original researchers via email to obtain any missing or inadequate data, if possible. If we cannot collect accurate data, we will exclude these studies.

#### 2.4.6 Assessment of heterogeneity

We will calculate the *I*^2^ statistic (*I*^2^ values of 0% to 40% being interpreted as “might not be important”; 30% to 60%: may represent moderate heterogeneity; 50% to 90%: may represent substantial heterogeneity; and 75% to 100%: represents considerable heterogeneity). We will use subgroup analysis to explore the causes of heterogeneity among the results of studies. Furthermore, meta-regression will be conducted using “metafor”^[27]^ package for R when there are more than 10 studies in the meta-analysis.

#### 2.4.7 Assessment of reporting biases

We will use the funnel plot and Egger test to assess publication bias if >10 articles are included.

#### 2.4.8 Data synthesis

The meta-analysis will be conducted using the Review Manager V.5.3 software. We will describe the effect size with RR for dichotomous data, and MD or SMD for continuous data. The Cochrane Handbook for Systematic Reviews of Interventions Version 6^[28]^ does not suggest that the choice between a fixed-effect and a random-effects meta-analysis based on a statistical test for heterogeneity. We will use fixed-effect models for the primary analyses, yet, we will also present random-effects estimates confirming the results.

#### 2.4.9 Subgroup analysis

If there is a sufficient number of RCTs for inclusion in the review, we plan to conduct a subgroup analysis to explore sources of heterogeneity. Subgroup analysis will be conducted based on the type of acupuncture (manual acupuncture or electroacupuncture), the testing time of secondary outcomes (3 or 7 days after intervention).

#### 2.4.10 Sensitivity analysis

We planned to conduct sensitivity analyses for the primary outcomes to confirm the robustness of our findings. To evaluate the internal validity of the studies or treatment adequacy, we will subsequently remove studies of “High risk” of bias, studies of “Some concerns” of bias using the “metafor” package, and level out function.

#### 2.4.11 Quality of the evidence

We will summarize the quality of evidence using the Grading of Recommendations Assessment, Development and Evaluation (GRADE) approach and present “Summary of findings” tables.^[29]^ The GRADE approach evaluates the quality of evidence as “high,” “moderate,” “low,” or “very low” by the outcome. The quality of evidence can be reduced by 5 factors (risk of bias, the inconsistency of results, indirectness of evidence, imprecision, and publication bias) and increase by 3 factors (large effect, dose response, opposing plausible residual bias, and confounding). Two review authors (JX, LW) will independently reduce or increase the quality of evidence and resolved disagreements by discussion.

## 3 Discussion

Sepsis is a severe life-threatening infection with organ dysfunction. It cost of 20.3 billion dollar or 5.2% of the total aggregate cost for all hospitalizations and was the most expensive condition treated according to Healthcare Cost and Utilization Project in 2011.^[6]^ Acupuncture is widely used for anti-inflammation may be related to reduce the levels of serum C-reactive protein (CRP) and procalcitonin (PCT).^[30,31]^ It also can regulate immunity by improve the ratio of T cells and immune cytokines. Acupuncture is simple operation and low cost, if the evidence could prove acupuncture is useful for sepsis, it might save some cost of sepsis and improve the quality of life. However, no systematic reviews on this topic have been published. We hope to evaluate the evidence from published RCTs for the effectiveness of acupuncture in treating sepsis.

## Author contributions

**Conceptualization**: Jin Xian, Ling Wang.

**Data curation**: Changyun Zhang, Yushuo Zhu, Jin Xian, Ling Wang.

**Formal analysis**: Jin Xian, Jian Wang.

**Methodology**: Xin Zhang, Qiwen Tan.

**Project administration**: Xin Zhang, Qiwen Tan.

**Supervision**: Huijuan Yu, Qiwen Tan.

**Writing – original draft**: Jin Xian, Ling Wang.

## Supplementary Material

Supplemental Digital Content
