# Supplementary material for: Efficacy and safety of acupuncture as a complementary therapy for sepsis: A protocol of systematic review and meta-analysis
Source: Medicine (Baltimore). 2019 Nov 27;98(48):e18025. doi: 10.1097/MD.0000000000018025 (PMC6890293; doi:10.1097/MD.0000000000018025)
Supplement: Supplemental Digital Content [file medi-98-e18025-s001.pdf]

Pubmed

#1 randomized controlled trial [pt]

#2 controlled clinical trial [pt]

#3 randomized [tiab]

#4 randomly [tiab]

#5 #1 OR #2 OR #3 OR #4

#6 acupuncture [MeSH]

#7 cupuncture [tiab]

#8 electroacupuncture [MeSH]

#9 electroacupuncture[tiab]

#10 electro-acupuncture [MeSH]

#11 electro-acupuncture [tiab]

#12 body acupuncture [tiab]

#13 #6 OR #7 OR #8 OR #9 OR #10 OR #11 OR #12

#14 sepsis [MeSH]

#15 sepsis [tiab]

#16 septic [MeSH]

#17 septic[tiab]

#18 #15 OR #16 OR #17

#19 #5 AND 13 AND #18

Embase

#1 'acupuncture'/exp OR acupuncture:ti,ab

#2 'electroacupuncture'/exp OR electroacupuncture:ti,ab

#3'electro-acupuncture':ti,ab

#4 OR #2 OR #3

#5 'sepsis'/exp OR sepsis:ti OR septic:ti

#6 #4 AND #5 AND ([controlled clinical trial]/lim OR [randomized controlled trial]/lim)

The Chorane library Central

#1 prophyla* [tw] OR prevent* [tw]

#2 migraine [tw]

#3 acupuncture [tw] OR electroacupuncture [tw]

#4 #1 AND #2 AND #3

China National Knowledge Infrastructure（CNKI）

#1 针刺 ti OR 电针 ti

#2 偏头痛 ti

#3 预防 ft OR 缓解期 ft

#4 随机对照 ft OR 对照 ft

#5 #1 AND #2 AND #3 AND #4

Chinese Biomedicine database（CBM）

#1 "针刺"[中文标题:智能]

#2 "预防"[全字段:智能]

#3“缓解期”[全字段:智能]

#4 #2 OR #3

#5 "偏头痛"[中文标题:智能]

#6 ("随机对照"[全字段:智能]) OR "对照"[全字段:智能]

#7 #1 AND #4 AND #5 AND #6

VIP database (VIP)

（（M=（针刺 OR 电针 OR 针灸 OR 毫针 OR 体针））AND M=（脓毒症） AND U=（随机） NOT T=（meta）

Wan Fang Database

(（ 主题:(针刺 OR 电针 OR 毫针 OR 针灸 OR 体针） AND 主题:(脓毒症) AND 全部:(随机) NOT 摘要:(大鼠)）)*Date:-2019

Traditional Chinese Medicine Literature Analysisand Retrieval Database（TCM）

(((((针刺[主题词] OR 电针[主题词]) OR 针灸[主题词]) OR 毫针[主题词]) OR 体针[主题词]) AND 脓毒症[主题词]) AND 随机[中文文摘]
